# *N*-(4-Chloro­benzo­yl)-4-methyl­benzene­sulfonamide

**DOI:** 10.1107/S1600536809055585

**Published:** 2010-01-09

**Authors:** P. A. Suchetan, B. Thimme Gowda, Sabine Foro, Hartmut Fuess

**Affiliations:** a Department of Chemistry, Mangalore University, Mangalagangotri 574 199, Mangalore, India; b Institute of Materials Science, Darmstadt University of Technology, Petersenstrasse 23, D-64287 Darmstadt, Germany

## Abstract

The asymmetric unit of the title compound, C_14_H_12_ClNO_3_S, contains two independent mol­ecules. The dihedral angles between the two aromatic rings in each mol­ecule are 81.0 (1) and 76.3 (1)°. In the crystal, mol­ecules are linked by N—H⋯O hydrogen bonds.

## Related literature

For background literature and similar structures, see: Gowda *et al.* (2009**a* ▶,b* ▶); Suchetan *et al.* (2009 ▶).
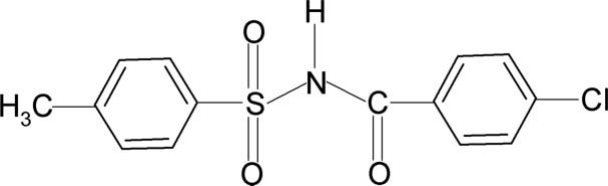


## Experimental

### 

#### Crystal data

C_14_H_12_ClNO_3_S
*M* *_r_* = 309.76
Monoclinic, 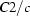

*a* = 25.675 (3) Å
*b* = 12.0508 (8) Å
*c* = 22.191 (3) Å
β = 122.16 (1)°
*V* = 5812.5 (11) Å^3^
*Z* = 16
Mo *K*α radiation
μ = 0.41 mm^−1^
*T* = 299 K
0.50 × 0.48 × 0.44 mm

#### Data collection

Oxford Diffraction Xcalibur diffractometer with a Sapphire CCD detector
Absorption correction: multi-scan (*CrysAlis RED*; Oxford Diffraction, 2009 ▶) *T*_min_ = 0.821, *T*_max_ = 0.840
12864 measured reflections
5931 independent reflections
3733 reflections with *I* > 2σ(*I*)
*R*_int_ = 0.018

#### Refinement

*R*[*F* ^2^ > 2σ(*F* ^2^)] = 0.045
*wR*(*F* ^2^) = 0.130
*S* = 1.07
5931 reflections
367 parameters
2 restraints
H atoms treated by a mixture of independent and constrained refinement
Δρ_max_ = 0.22 e Å^−3^
Δρ_min_ = −0.35 e Å^−3^

### 

Data collection: *CrysAlis CCD* (Oxford Diffraction, 2009 ▶); cell refinement: *CrysAlis RED* (Oxford Diffraction, 2009 ▶); data reduction: *CrysAlis RED*; program(s) used to solve structure: *SHELXS97* (Sheldrick, 2008 ▶); program(s) used to refine structure: *SHELXL97* (Sheldrick, 2008 ▶); molecular graphics: *PLATON* (Spek, 2009 ▶); software used to prepare material for publication: *SHELXL97*.

## Supplementary Material

Crystal structure: contains datablocks I, global. DOI: 10.1107/S1600536809055585/bt5156sup1.cif

Structure factors: contains datablocks I. DOI: 10.1107/S1600536809055585/bt5156Isup2.hkl

Additional supplementary materials:  crystallographic information; 3D view; checkCIF report

## Figures and Tables

**Table 1:** Hydrogen-bond geometry (Å, °)

*D*—H⋯*A*	*D*—H	H⋯*A*	*D*⋯*A*	*D*—H⋯*A*
N1—H1*N*⋯O5^i^	0.84 (1)	2.35 (1)	3.133 (3)	156 (2)
N2—H2*N*⋯O2^ii^	0.87 (1)	2.03 (1)	2.890 (3)	170 (2)

Symmetry codes: (i) 
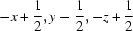
; (ii) 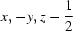.

**Fig. 1.**
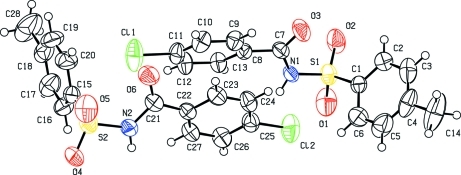
Molecular structure of (I), showing the atom labelling scheme and the displacement ellipsoids are drawn at the 50% probability level.

**Fig. 2.**
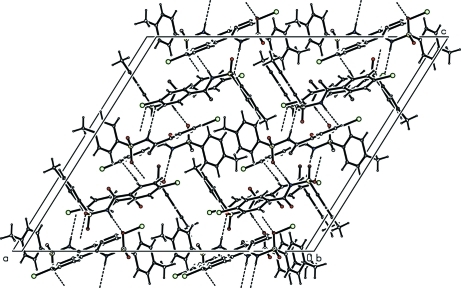
Molecular packing of (I) with hydrogen bonding shown as dashed lines.

## supplementary crystallographic information

### Comment

Diaryl acylsulfonamides are known as potent antitumor agents against a broad spectrum of human tumor xenografts in nude mice. As part of a study of the effect of ring and the side chain substituents on the crystal structures of *N*-aromatic sulfonamides (Gowda *et al.*, 2009*a,b*; Suchetan *et al.*, 2009), in the present work, the structure of *N*-(4-chlorobenzoyl)4-methylbenzenesulfonamide (I) has been determined (Fig.1). The conformations of the N—H bonds in the C—SO_2_—NH—C(O) segments of the structure are *anti* to the C=O bonds, similar to that observed in *N*-(benzoyl)benzenesulfonamide (II) (Gowda *et al.*, 2009*a*) and *N*-(4-chlorobenzoyl) benzenesulfonamide (III)(Suchetan *et al.*, 2009).

The molecules are twisted at the *S* atom with the torsional angles of 67.1 (2)° and 67.7 (2)°, in the two molecules. The dihedral angles between the sulfonyl benzene ring and the —SO_2_—NH—C—O segment are 83.6 (1)° and 81.0 (1)°, compared to the values of 86.5(0.1) in (II) and 75.7 (1)° in (III). Furthermore, the dihedral angle between the sulfonyl and the benzoyl benzene rings in (I) are 81.0 (1)° and 76.3 (1)°, compared to the values of 80.3(0.1) in (II) and 68.6 (1)° in (III).

The dihedral angle between the sulfonyl benzene rings of the two molecules in the asymmetric unit is 45.8 (1)°. The packing of molecules linked by of N—H···O(S) hydrogen bonds (Table 1) is shown in Fig. 2.

### Experimental

*N*-(4-Chlorobenzoyl)4-methylbenzenesulfonamide was prepared by heating a mixture of 4-methylbenzenesulfonamide and 4-chlorobenzoyl chloride at 60° C for one hour. The reaction mixture was cooled and poured into ice cold water. The resulting solid was separated, washed thoroughly with water and dissolved in sodium hydrogen carbonate solution. The compound was precipitated by acidifying the filtered solution with dil. HCl. It was filtered and dried. The purity of the compound was checked by recording its melting point (168–170° C). Single crystals were obtained from slow evaporation of a solution of the compound in toluene. Prism like colourless single crystals of the title compound were obtained from a slow evaporation of its toluene solution at room temperature and the X-ray diffraction studies were also carried out at room temperature.

### Refinement

The H atoms of the NH groups were located in a difference map and later restrained to N—H = 0.86 (1) %A. The other H atoms were positioned with idealized geometry using a riding model with C—H = 0.93 Å All H atoms were refined with isotropic displacement parameters (set to 1.2 times of the *U*_eq_ of the parent atom).

### Crystal data

**Table:**

C_14_H_12_ClNO_3_S	*F*(000) = 2560
*M**_r_* = 309.76	*D*_x_ = 1.416 Mg m^−^^3^
Monoclinic, *C*2/*c*	Mo *K*α radiation, λ = 0.71073 Å
Hall symbol: -C 2yc	Cell parameters from 3720 reflections
*a* = 25.675 (3) Å	θ = 2.4–27.8°
*b* = 12.0508 (8) Å	µ = 0.41 mm^−^^1^
*c* = 22.191 (3) Å	*T* = 299 K
β = 122.16 (1)°	Prism, colourless
*V* = 5812.5 (11) Å^3^	0.50 × 0.48 × 0.44 mm
*Z* = 16	

### Data collection

**Table:**

Oxford Diffraction Xcalibur diffractometer with a Sapphire CCD detector	5931 independent reflections
Radiation source: fine-focus sealed tube	3733 reflections with *I* > 2σ(*I*)
graphite	*R*_int_ = 0.018
Rotation method data acquisition using ω and phi scans	θ_max_ = 26.4°, θ_min_ = 2.5°
Absorption correction: multi-scan (*CrysAlis RED*; Oxford Diffraction, 2009)	*h* = −25→32
*T*_min_ = 0.821, *T*_max_ = 0.840	*k* = −10→15
12864 measured reflections	*l* = −27→26

### Refinement

**Table:**

Refinement on *F*^2^	Primary atom site location: structure-invariant direct methods
Least-squares matrix: full	Secondary atom site location: difference Fourier map
*R*[*F*^2^ > 2σ(*F*^2^)] = 0.045	Hydrogen site location: inferred from neighbouring sites
*wR*(*F*^2^) = 0.130	H atoms treated by a mixture of independent and constrained refinement
*S* = 1.07	*w* = 1/[σ^2^(*F*_o_^2^) + (0.0686*P*)^2^ + 0.2769*P*] where *P* = (*F*_o_^2^ + 2*F*_c_^2^)/3
5931 reflections	(Δ/σ)_max_ < 0.001
367 parameters	Δρ_max_ = 0.22 e Å^−^^3^
2 restraints	Δρ_min_ = −0.35 e Å^−^^3^

### Special details

**Table:**

Experimental. CrysAlis RED (Oxford Diffraction, 2009) Empirical absorption correction using spherical harmonics, implemented in SCALE3 ABSPACK scaling algorithm.
Geometry. All e.s.d.'s (except the e.s.d. in the dihedral angle between two l.s. planes) are estimated using the full covariance matrix. The cell e.s.d.'s are taken into account individually in the estimation of e.s.d.'s in distances, angles and torsion angles; correlations between e.s.d.'s in cell parameters are only used when they are defined by crystal symmetry. An approximate (isotropic) treatment of cell e.s.d.'s is used for estimating e.s.d.'s involving l.s. planes.
Refinement. Refinement of *F*^2^ against ALL reflections. The weighted *R*-factor *wR* and goodness of fit *S* are based on *F*^2^, conventional *R*-factors *R* are based on *F*, with *F* set to zero for negative *F*^2^. The threshold expression of *F*^2^ > σ(*F*^2^) is used only for calculating *R*-factors(gt) *etc*. and is not relevant to the choice of reflections for refinement. *R*-factors based on *F*^2^ are statistically about twice as large as those based on *F*, and *R*- factors based on ALL data will be even larger.

### Fractional atomic coordinates and isotropic or equivalent isotropic displacement parameters (Å^2^)

**Table:**

	*x*	*y*	*z*	*U*_iso_*/*U*_eq_	
Cl1	0.38789 (4)	0.05434 (7)	0.38377 (5)	0.0983 (3)	
S1	0.14332 (3)	−0.10122 (5)	0.50954 (3)	0.05366 (19)	
O1	0.11216 (9)	−0.19893 (15)	0.47163 (12)	0.0881 (6)	
O2	0.18104 (8)	−0.10514 (17)	0.58521 (9)	0.0788 (6)	
O3	0.22829 (9)	0.08559 (16)	0.54283 (10)	0.0806 (6)	
N1	0.18659 (8)	−0.06886 (16)	0.47816 (9)	0.0452 (5)	
H1N	0.1773 (10)	−0.1095 (16)	0.4431 (9)	0.054*	
C1	0.09034 (10)	0.00583 (18)	0.48616 (12)	0.0442 (5)	
C2	0.09089 (11)	0.0707 (2)	0.53769 (14)	0.0585 (7)	
H2	0.1209	0.0605	0.5855	0.070*	
C3	0.04631 (14)	0.1506 (2)	0.51720 (18)	0.0740 (8)	
H3	0.0462	0.1941	0.5518	0.089*	
C4	0.00175 (14)	0.1678 (3)	0.4465 (2)	0.0793 (9)	
C5	0.00290 (14)	0.1015 (3)	0.39662 (17)	0.0886 (10)	
H5	−0.0269	0.1118	0.3487	0.106*	
C6	0.04580 (12)	0.0223 (2)	0.41515 (14)	0.0691 (7)	
H6	0.0455	−0.0211	0.3803	0.083*	
C7	0.22739 (10)	0.01850 (19)	0.50176 (12)	0.0477 (6)	
C8	0.26807 (10)	0.02355 (18)	0.47333 (11)	0.0417 (5)	
C9	0.28899 (10)	0.12670 (19)	0.46694 (13)	0.0532 (6)	
H9	0.2778	0.1901	0.4812	0.064*	
C10	0.32578 (11)	0.1362 (2)	0.44001 (13)	0.0566 (7)	
H10	0.3397	0.2055	0.4361	0.068*	
C11	0.34187 (11)	0.0432 (2)	0.41897 (13)	0.0547 (6)	
C12	0.32329 (11)	−0.0603 (2)	0.42626 (13)	0.0580 (7)	
H12	0.3360	−0.1233	0.4133	0.070*	
C13	0.28596 (10)	−0.06994 (19)	0.45281 (12)	0.0494 (6)	
H13	0.2726	−0.1396	0.4570	0.059*	
C14	−0.04607 (16)	0.2566 (3)	0.4263 (2)	0.1319 (16)	
H14A	−0.0263	0.3246	0.4500	0.158*	
H14B	−0.0740	0.2341	0.4402	0.158*	
H14C	−0.0684	0.2679	0.3757	0.158*	
Cl2	0.10332 (4)	0.14098 (7)	0.31950 (5)	0.0936 (3)	
S2	0.33983 (3)	0.23886 (6)	0.17178 (3)	0.0584 (2)	
O4	0.31253 (8)	0.18435 (17)	0.10439 (8)	0.0764 (6)	
O5	0.35779 (10)	0.35126 (16)	0.17631 (11)	0.0853 (6)	
O6	0.33220 (8)	0.33394 (15)	0.29154 (9)	0.0688 (5)	
N2	0.28714 (9)	0.22737 (16)	0.19241 (10)	0.0510 (5)	
H2N	0.2582 (8)	0.1838 (16)	0.1619 (10)	0.061*	
C15	0.40193 (10)	0.1613 (2)	0.23652 (12)	0.0501 (6)	
C16	0.39912 (11)	0.0479 (2)	0.23028 (13)	0.0613 (7)	
H16	0.3644	0.0142	0.1926	0.074*	
C17	0.44735 (13)	−0.0156 (3)	0.27945 (16)	0.0755 (8)	
H17	0.4450	−0.0924	0.2745	0.091*	
C18	0.49838 (13)	0.0307 (4)	0.33503 (16)	0.0806 (9)	
C19	0.50120 (13)	0.1440 (4)	0.34170 (15)	0.0901 (11)	
H19	0.5361	0.1766	0.3797	0.108*	
C20	0.45307 (13)	0.2109 (3)	0.29301 (14)	0.0734 (8)	
H20	0.4552	0.2876	0.2984	0.088*	
C21	0.29100 (11)	0.27162 (19)	0.25229 (12)	0.0486 (6)	
C22	0.24186 (10)	0.23757 (18)	0.26438 (11)	0.0433 (5)	
C23	0.22642 (11)	0.3098 (2)	0.30130 (12)	0.0533 (6)	
H23	0.2454	0.3788	0.3154	0.064*	
C24	0.18339 (12)	0.2803 (2)	0.31707 (13)	0.0585 (7)	
H24	0.1725	0.3296	0.3408	0.070*	
C25	0.15648 (11)	0.1773 (2)	0.29750 (13)	0.0555 (6)	
C26	0.17150 (12)	0.1034 (2)	0.26192 (13)	0.0576 (6)	
H26	0.1531	0.0338	0.2490	0.069*	
C27	0.21412 (11)	0.13404 (19)	0.24571 (12)	0.0517 (6)	
H27	0.2246	0.0844	0.2218	0.062*	
C28	0.55230 (14)	−0.0392 (4)	0.38905 (19)	0.1392 (17)	
H28A	0.5748	−0.0653	0.3685	0.167*	
H28B	0.5374	−0.1014	0.4026	0.167*	
H28C	0.5789	0.0050	0.4303	0.167*	

### Atomic displacement parameters (Å^2^)

**Table:**

	*U*^11^	*U*^22^	*U*^33^	*U*^12^	*U*^13^	*U*^23^
Cl1	0.1146 (7)	0.0990 (6)	0.1408 (8)	0.0175 (5)	0.1081 (6)	0.0329 (5)
S1	0.0629 (4)	0.0522 (4)	0.0623 (4)	0.0014 (3)	0.0443 (3)	0.0062 (3)
O1	0.1045 (15)	0.0493 (11)	0.1475 (19)	−0.0261 (11)	0.0920 (15)	−0.0236 (12)
O2	0.0802 (12)	0.1132 (16)	0.0579 (12)	0.0377 (12)	0.0467 (10)	0.0363 (11)
O3	0.0916 (14)	0.0817 (14)	0.0988 (15)	−0.0321 (11)	0.0711 (13)	−0.0506 (12)
N1	0.0521 (11)	0.0500 (12)	0.0439 (11)	−0.0115 (9)	0.0326 (10)	−0.0143 (9)
C1	0.0444 (12)	0.0488 (14)	0.0459 (14)	−0.0055 (10)	0.0283 (11)	0.0032 (10)
C2	0.0564 (15)	0.0642 (17)	0.0577 (16)	−0.0022 (13)	0.0323 (13)	−0.0058 (13)
C3	0.080 (2)	0.0623 (19)	0.107 (3)	0.0012 (16)	0.069 (2)	−0.0054 (17)
C4	0.0615 (18)	0.074 (2)	0.124 (3)	0.0162 (16)	0.064 (2)	0.042 (2)
C5	0.0615 (18)	0.129 (3)	0.074 (2)	0.020 (2)	0.0349 (17)	0.041 (2)
C6	0.0667 (17)	0.091 (2)	0.0517 (17)	0.0017 (16)	0.0326 (14)	0.0052 (15)
C7	0.0526 (14)	0.0490 (14)	0.0448 (14)	−0.0029 (12)	0.0281 (11)	−0.0081 (11)
C8	0.0420 (12)	0.0412 (13)	0.0431 (13)	−0.0006 (10)	0.0233 (10)	−0.0018 (10)
C9	0.0556 (14)	0.0412 (14)	0.0663 (16)	0.0040 (11)	0.0348 (13)	−0.0005 (11)
C10	0.0555 (15)	0.0457 (15)	0.0730 (18)	0.0020 (12)	0.0372 (14)	0.0153 (12)
C11	0.0552 (14)	0.0602 (17)	0.0630 (16)	0.0089 (13)	0.0411 (13)	0.0166 (13)
C12	0.0678 (16)	0.0494 (15)	0.0771 (18)	0.0106 (13)	0.0522 (15)	0.0037 (13)
C13	0.0585 (14)	0.0401 (14)	0.0625 (16)	−0.0003 (11)	0.0408 (13)	0.0012 (11)
C14	0.101 (3)	0.113 (3)	0.227 (5)	0.051 (2)	0.118 (3)	0.081 (3)
Cl2	0.0976 (6)	0.1036 (7)	0.1202 (7)	−0.0049 (5)	0.0852 (6)	0.0033 (5)
S2	0.0639 (4)	0.0694 (5)	0.0533 (4)	−0.0019 (3)	0.0388 (3)	0.0103 (3)
O4	0.0730 (12)	0.1215 (16)	0.0424 (10)	0.0038 (12)	0.0358 (9)	−0.0008 (10)
O5	0.1050 (15)	0.0662 (13)	0.1101 (17)	−0.0067 (11)	0.0744 (14)	0.0229 (11)
O6	0.0759 (12)	0.0704 (12)	0.0660 (12)	−0.0237 (10)	0.0417 (10)	−0.0165 (10)
N2	0.0519 (12)	0.0614 (14)	0.0430 (11)	−0.0046 (10)	0.0275 (10)	−0.0021 (9)
C15	0.0492 (14)	0.0624 (17)	0.0465 (14)	−0.0135 (12)	0.0307 (12)	−0.0030 (12)
C16	0.0503 (15)	0.0705 (19)	0.0578 (17)	−0.0067 (14)	0.0252 (13)	−0.0029 (14)
C17	0.0630 (18)	0.082 (2)	0.080 (2)	0.0059 (16)	0.0372 (18)	0.0178 (17)
C18	0.0546 (18)	0.127 (3)	0.064 (2)	0.000 (2)	0.0343 (16)	0.024 (2)
C19	0.0490 (17)	0.157 (4)	0.0515 (19)	−0.036 (2)	0.0178 (15)	−0.010 (2)
C20	0.0704 (18)	0.087 (2)	0.0611 (18)	−0.0333 (17)	0.0337 (16)	−0.0167 (16)
C21	0.0584 (15)	0.0430 (14)	0.0453 (14)	0.0013 (12)	0.0281 (12)	0.0025 (11)
C22	0.0521 (13)	0.0399 (13)	0.0380 (12)	0.0053 (11)	0.0241 (11)	0.0052 (10)
C23	0.0672 (16)	0.0379 (13)	0.0566 (15)	−0.0008 (12)	0.0341 (13)	−0.0041 (11)
C24	0.0702 (17)	0.0538 (16)	0.0626 (17)	0.0121 (14)	0.0428 (14)	−0.0011 (13)
C25	0.0586 (15)	0.0609 (17)	0.0552 (15)	0.0058 (13)	0.0358 (13)	0.0081 (13)
C26	0.0703 (16)	0.0476 (15)	0.0643 (16)	−0.0079 (13)	0.0421 (14)	−0.0018 (13)
C27	0.0667 (16)	0.0457 (14)	0.0510 (15)	0.0020 (12)	0.0369 (13)	−0.0026 (11)
C28	0.069 (2)	0.233 (5)	0.096 (3)	0.032 (3)	0.030 (2)	0.081 (3)

### Geometric parameters (Å, °)

**Table:**

Cl1—C11	1.734 (2)	Cl2—C25	1.732 (2)
S1—O1	1.4205 (19)	S2—O5	1.4170 (19)
S1—O2	1.4242 (18)	S2—O4	1.4294 (18)
S1—N1	1.6417 (18)	S2—N2	1.6487 (19)
S1—C1	1.741 (2)	S2—C15	1.744 (2)
O3—C7	1.209 (2)	O6—C21	1.209 (3)
N1—C7	1.377 (3)	N2—C21	1.385 (3)
N1—H1N	0.838 (9)	N2—H2N	0.867 (9)
C1—C2	1.379 (3)	C15—C16	1.371 (3)
C1—C6	1.383 (3)	C15—C20	1.378 (3)
C2—C3	1.375 (3)	C16—C17	1.368 (4)
C2—H2	0.9300	C16—H16	0.9300
C3—C4	1.380 (4)	C17—C18	1.352 (4)
C3—H3	0.9300	C17—H17	0.9300
C4—C5	1.377 (5)	C18—C19	1.371 (4)
C4—C14	1.507 (4)	C18—C28	1.515 (4)
C5—C6	1.345 (4)	C19—C20	1.387 (4)
C5—H5	0.9300	C19—H19	0.9300
C6—H6	0.9300	C20—H20	0.9300
C7—C8	1.481 (3)	C21—C22	1.481 (3)
C8—C13	1.382 (3)	C22—C27	1.386 (3)
C8—C9	1.391 (3)	C22—C23	1.390 (3)
C9—C10	1.365 (3)	C23—C24	1.372 (3)
C9—H9	0.9300	C23—H23	0.9300
C10—C11	1.361 (3)	C24—C25	1.374 (3)
C10—H10	0.9300	C24—H24	0.9300
C11—C12	1.375 (3)	C25—C26	1.374 (3)
C12—C13	1.371 (3)	C26—C27	1.372 (3)
C12—H12	0.9300	C26—H26	0.9300
C13—H13	0.9300	C27—H27	0.9300
C14—H14A	0.9600	C28—H28A	0.9600
C14—H14B	0.9600	C28—H28B	0.9600
C14—H14C	0.9600	C28—H28C	0.9600
			
O1—S1—O2	119.07 (13)	O5—S2—O4	118.25 (12)
O1—S1—N1	103.93 (10)	O5—S2—N2	110.16 (11)
O2—S1—N1	108.60 (10)	O4—S2—N2	103.53 (10)
O1—S1—C1	109.47 (12)	O5—S2—C15	109.49 (12)
O2—S1—C1	108.00 (11)	O4—S2—C15	109.86 (12)
N1—S1—C1	107.18 (10)	N2—S2—C15	104.58 (10)
C7—N1—S1	124.74 (15)	C21—N2—S2	125.70 (17)
C7—N1—H1N	125.6 (16)	C21—N2—H2N	125.2 (16)
S1—N1—H1N	109.4 (16)	S2—N2—H2N	108.9 (16)
C2—C1—C6	120.0 (2)	C16—C15—C20	119.8 (3)
C2—C1—S1	120.74 (19)	C16—C15—S2	118.36 (18)
C6—C1—S1	119.16 (19)	C20—C15—S2	121.8 (2)
C3—C2—C1	118.9 (3)	C17—C16—C15	120.0 (3)
C3—C2—H2	120.5	C17—C16—H16	120.0
C1—C2—H2	120.5	C15—C16—H16	120.0
C2—C3—C4	121.4 (3)	C18—C17—C16	121.5 (3)
C2—C3—H3	119.3	C18—C17—H17	119.2
C4—C3—H3	119.3	C16—C17—H17	119.2
C5—C4—C3	117.9 (3)	C17—C18—C19	118.6 (3)
C5—C4—C14	122.4 (4)	C17—C18—C28	121.8 (4)
C3—C4—C14	119.7 (4)	C19—C18—C28	119.6 (3)
C6—C5—C4	122.0 (3)	C18—C19—C20	121.4 (3)
C6—C5—H5	119.0	C18—C19—H19	119.3
C4—C5—H5	119.0	C20—C19—H19	119.3
C5—C6—C1	119.8 (3)	C15—C20—C19	118.6 (3)
C5—C6—H6	120.1	C15—C20—H20	120.7
C1—C6—H6	120.1	C19—C20—H20	120.7
O3—C7—N1	120.2 (2)	O6—C21—N2	121.2 (2)
O3—C7—C8	124.0 (2)	O6—C21—C22	123.0 (2)
N1—C7—C8	115.83 (18)	N2—C21—C22	115.7 (2)
C13—C8—C9	118.7 (2)	C27—C22—C23	118.5 (2)
C13—C8—C7	122.7 (2)	C27—C22—C21	123.2 (2)
C9—C8—C7	118.6 (2)	C23—C22—C21	118.1 (2)
C10—C9—C8	121.0 (2)	C24—C23—C22	120.6 (2)
C10—C9—H9	119.5	C24—C23—H23	119.7
C8—C9—H9	119.5	C22—C23—H23	119.7
C11—C10—C9	119.2 (2)	C23—C24—C25	119.4 (2)
C11—C10—H10	120.4	C23—C24—H24	120.3
C9—C10—H10	120.4	C25—C24—H24	120.3
C10—C11—C12	121.3 (2)	C26—C25—C24	121.3 (2)
C10—C11—Cl1	119.64 (19)	C26—C25—Cl2	120.2 (2)
C12—C11—Cl1	119.06 (19)	C24—C25—Cl2	118.56 (19)
C13—C12—C11	119.5 (2)	C27—C26—C25	118.9 (2)
C13—C12—H12	120.2	C27—C26—H26	120.5
C11—C12—H12	120.2	C25—C26—H26	120.5
C12—C13—C8	120.2 (2)	C26—C27—C22	121.2 (2)
C12—C13—H13	119.9	C26—C27—H27	119.4
C8—C13—H13	119.9	C22—C27—H27	119.4
C4—C14—H14A	109.5	C18—C28—H28A	109.5
C4—C14—H14B	109.5	C18—C28—H28B	109.5
H14A—C14—H14B	109.5	H28A—C28—H28B	109.5
C4—C14—H14C	109.5	C18—C28—H28C	109.5
H14A—C14—H14C	109.5	H28A—C28—H28C	109.5
H14B—C14—H14C	109.5	H28B—C28—H28C	109.5
			
O1—S1—N1—C7	−177.1 (2)	O5—S2—N2—C21	−49.9 (2)
O2—S1—N1—C7	−49.4 (2)	O4—S2—N2—C21	−177.2 (2)
C1—S1—N1—C7	67.1 (2)	C15—S2—N2—C21	67.7 (2)
O1—S1—C1—C2	132.2 (2)	O5—S2—C15—C16	−164.32 (18)
O2—S1—C1—C2	1.2 (2)	O4—S2—C15—C16	−32.9 (2)
N1—S1—C1—C2	−115.67 (19)	N2—S2—C15—C16	77.7 (2)
O1—S1—C1—C6	−45.1 (2)	O5—S2—C15—C20	15.6 (2)
O2—S1—C1—C6	−176.12 (19)	O4—S2—C15—C20	147.08 (19)
N1—S1—C1—C6	67.0 (2)	N2—S2—C15—C20	−102.4 (2)
C6—C1—C2—C3	0.6 (4)	C20—C15—C16—C17	−1.0 (4)
S1—C1—C2—C3	−176.69 (19)	S2—C15—C16—C17	179.0 (2)
C1—C2—C3—C4	−0.7 (4)	C15—C16—C17—C18	0.3 (4)
C2—C3—C4—C5	0.5 (4)	C16—C17—C18—C19	0.1 (4)
C2—C3—C4—C14	−179.7 (2)	C16—C17—C18—C28	−179.1 (3)
C3—C4—C5—C6	−0.2 (4)	C17—C18—C19—C20	0.2 (5)
C14—C4—C5—C6	180.0 (3)	C28—C18—C19—C20	179.4 (3)
C4—C5—C6—C1	0.1 (4)	C16—C15—C20—C19	1.2 (4)
C2—C1—C6—C5	−0.3 (4)	S2—C15—C20—C19	−178.8 (2)
S1—C1—C6—C5	177.0 (2)	C18—C19—C20—C15	−0.8 (4)
S1—N1—C7—O3	−8.7 (3)	S2—N2—C21—O6	8.0 (3)
S1—N1—C7—C8	171.77 (16)	S2—N2—C21—C22	−172.02 (15)
O3—C7—C8—C13	151.8 (3)	O6—C21—C22—C27	−147.2 (2)
N1—C7—C8—C13	−28.6 (3)	N2—C21—C22—C27	32.8 (3)
O3—C7—C8—C9	−28.3 (4)	O6—C21—C22—C23	27.5 (3)
N1—C7—C8—C9	151.2 (2)	N2—C21—C22—C23	−152.5 (2)
C13—C8—C9—C10	0.9 (3)	C27—C22—C23—C24	−1.8 (3)
C7—C8—C9—C10	−179.0 (2)	C21—C22—C23—C24	−176.8 (2)
C8—C9—C10—C11	0.2 (4)	C22—C23—C24—C25	1.5 (4)
C9—C10—C11—C12	−1.9 (4)	C23—C24—C25—C26	−0.5 (4)
C9—C10—C11—Cl1	179.30 (18)	C23—C24—C25—Cl2	178.81 (18)
C10—C11—C12—C13	2.4 (4)	C24—C25—C26—C27	−0.2 (4)
Cl1—C11—C12—C13	−178.81 (18)	Cl2—C25—C26—C27	−179.45 (17)
C11—C12—C13—C8	−1.2 (4)	C25—C26—C27—C22	−0.2 (4)
C9—C8—C13—C12	−0.4 (3)	C23—C22—C27—C26	1.2 (3)
C7—C8—C13—C12	179.4 (2)	C21—C22—C27—C26	175.9 (2)

### Hydrogen-bond geometry (Å, °)

**Table:**

*D*—H···*A*	*D*—H	H···*A*	*D*···*A*	*D*—H···*A*
N1—H1N···O5^i^	0.84 (1)	2.35 (1)	3.133 (3)	156 (2)
N2—H2N···O2^ii^	0.87 (1)	2.03 (1)	2.890 (3)	170 (2)

Symmetry codes: (i) −*x*+1/2, *y*−1/2, −*z*+1/2; (ii) *x*, −*y*, *z*−1/2.
